# Identification and characterization of Pv50, a novel *Plasmodium vivax* merozoite surface protein

**DOI:** 10.1186/s13071-019-3434-7

**Published:** 2019-04-18

**Authors:** Yang Cheng, Bo Wang, Feng Lu, Md Atique Ahmed, Jin-Hee Han, Sung Hun Na, Kwon-Soo Ha, Won Sun Park, Seok-Ho Hong, Eun-Taek Han

**Affiliations:** 1 Department of Public Health and Preventive Medicine, Laboratory of Pathogen Infection and Immunity, Wuxi School of Medicine, Jiangnan University, Wuxi, Jiangsu People’s Republic of China; 2 Department of Medical Environmental Biology and Tropical Medicine, School of Medicine, Kangwon National University, Chuncheon, Gangwon-do Republic of Korea; 3 Department of Clinical Laboratory, The First Affiliated Hospital of Anhui Medical University, Hefei, Anhui People’s Republic of China; 4 Department of Pathogen Biology and Immunology, School of Medicine, Yangzhou University, Yangzhou, People’s Republic of China; 5 Department of Obstetrics and Gynecology, Kangwon National University Hospital, Kangwon National University School of Medicine, Chuncheon, 24341 South Korea; 6 Department of Molecular and Cellular Biochemistry, School of Medicine, Kangwon National University, Chuncheon, Gangwon-do 24341 Republic of Korea; 7 Department of Physiology, School of Medicine, Kangwon National University, Chuncheon, Gangwon-do 24341 Republic of Korea; 8 Department of Internal Medicine, School of Medicine, Kangwon National University, Chuncheon, Gangwon-do 24341 Republic of Korea

**Keywords:** *Plasmodium vivax*, Merozoite surface protein, Pv50, Protein interaction, Antigenicity, Immunogenicity

## Abstract

**Background:**

*Plasmodium vivax* contains approximately 5400 coding genes, more than 40% of which code for hypothetical proteins that have not been functionally characterized. In a previous preliminary screening using pooled serum samples, numerous hypothetical proteins were selected from among those that were highly transcribed in the schizont-stage of parasites, and highly antigenic *P. vivax* candidates including hypothetical proteins were identified. However, their immunological and functional activities in *P. vivax* remain unclear. From these candidates, we investigated a *P. vivax* 50-kDa protein (Pv50, PVX_087140) containing a highly conserved signal peptide that shows high transcription levels in blood-stage parasites.

**Results:**

Recombinant Pv50 was expressed in a cell-free expression system and used for IgG prevalence analysis of patients with vivax malaria and healthy individuals. Immune responses were analyzed in immunized mice and mouse antibodies were used to detect the subcellular localization of the protein in blood-stage parasites by immunofluorescence assay. A protein array method was used to evaluate protein-protein interactions to predict protein functional activities during the invasion of parasites into erythrocytes. Recombinant Pv50 showed IgG prevalence in patient samples with a sensitivity of 42.9% and specificity of 93.8% compared to that in healthy individuals. The non-cytophilic antibodies IgG1 and IgG3 were the major components involved in the antibody response in Pv50-immunized mice. Pv50 localized on the surface of merozoites and a specific interaction between Pv50 and PvMSP1 was detected, suggesting that Pv50-PvMSP1 forms a heterodimeric complex in *P. vivax*.

**Conclusions:**

Increased immune responses caused by native *P. vivax* parasites were detected, confirming its immunogenic effects. This study provides a method for detecting new malaria antigens, and Pv50 may be a vivax malaria vaccine candidate with PvMSP1.

**Electronic supplementary material:**

The online version of this article (10.1186/s13071-019-3434-7) contains supplementary material, which is available to authorized users.

## Background

Although infection by *Plasmodium vivax* has been referred to as “benign tertian malaria”, it represents a major threat to health in South Asia, Southeast Asia, South America and even in Africa; *P. vivax* malaria has caused an estimated 8.5 million annual infections and 3100 deaths [1–3]. Currently, no effective vaccines for preventing malaria are available. *Plasmodium vivax* has been neglected as a tropical disease [4], and only two *P. vivax* vaccine candidates are in preliminary (phase I) clinical trials [5]. Additionally, *P. vivax* contains numerous unknown antigens involved in malaria pathobiology. Understanding the function and characteristics of hypothetical proteins in *Plasmodium* is important for developing a malaria vaccine.

In *Plasmodium* parasite pathobiology, invasion and subsequent modification of human erythrocytes are considered essential processes [6]. During invasion, most potential proteins are either secreted from the apical organelles or located on the merozoite surface. On the parasite membrane surface, some glycosylphosphatidylinositol (GPI)-anchored merozoite surface proteins anchor into the merozoite surface when the GPI-motif combines with some non-covalently associated proteins (MSP6, MSP7 and Pf41) [7–9]. Additionally, merozoite surface proteins are largely comprised of GPI-anchored membrane proteins and their associated partners are considered to directly induce the host immune response.

Antibodies likely play a crucial role in host protection through several mechanisms such as inhibition of parasite invasion, intraerythrocytic parasite blockage and mononuclear cell-mediated inhibition [10]. As a hypothetical and conserved protein, Pv50 (PVX_087140) was identified in *P. vivax* blood stage antigens and found to react with *P. vivax* patients’ pooled serum samples in a previous preliminary screening study [11]. To characterize the hypothetical protein Pv50, its immunogenic profiles, humoral and cellular immune responses, protein interactions and subcellular localization in parasites were evaluated. We expressed and purified a constructed Pv50 protein based on the Sal-I strain sequence using a wheat germ cell-free expression system [12]. Analysis of the human immune response against this purified Pv50 using 112 vivax patient samples and 80 healthy samples revealed that Pv50 possessed high antigenicity. We measured the levels of antibodies (total IgG and IgG subclasses) specifically against Pv50 from patients infected with vivax and immunized mice. Furthermore, the cellular immune response was measured and cytokine levels in immunized mice splenocytes were measured. According to the localization of Pv50, a protein-protein interaction was identified between Pv50 and PvMSP1.

## Methods

### Gene identification and protein sequence analysis

The sequence data and gene expression profiles of *pv50* (accession no. PVX_087140) were retrieved from and analyzed with PlasmoDB (http://plasmoDB.org). Predicted protein domains were further analyzed using the Simple Modular Architecture Research Tool (http://smart.embl-heidelberg.de/) and SOSUIsignal (http://bp.nuap.nagoya-u.ac.jp/sosui/). The orthologue sequences of *pv50* were obtained from PlasmoDB and a phylogenic tree was generated using DNAstar MegAlign software V7.2 (DNASTAR, Madison, WI, USA) with the Clustal V method. Sixty-four worldwide isolate sequences from seven countries including China (*n* = 5), Columbia (*n* = 20), India (*n* = 1), Mexico (*n* = 14), Peru (*n* = 14), Papua New Guinea (*n* = 2) and Thailand (*n* = 8) were obtained from PlasmoDB. Sequence diversity (π) analysis was performed and visualized using DNAsp v.5.0 software with a sliding window length of 25 sites and step size of 5 sites.

### Human sera samples

At local hospitals and clinics in endemic areas of the Republic of Korea (ROK), sera samples were collected from 112 patients positive for vivax malaria (mean age, 24.8 years; range, 18–42 years); patients were confirmed to be symptom- and parasite-positive (mean parasitemia, 0.121%; range, 0.010–0.46%) by microscopy. Eighty healthy individual sera samples were collected from non-endemic areas of the ROK and used in this study.

### Expression and purification of recombinant Pv50

Recombinant Pv50 was designed based on the *P. vivax* Sal-I strain sequence (PlasmoDB PVX_087140) and amplified from genomic DNA of *P. vivax* isolates from the ROK. Genomic DNA was prepared as described previously [13]. The coding gene for Pv50 was amplified using genomic DNA with the In-fusion cloning primers Pv50-F (5′-GGG CGG ATA TCT CGA GAA CTT TTT CAC CTC CTG CTC TG-3′) and Pv50-R (5′-GCG GTA CCC GGG ATC CTC ACT TCC TCT TCG CTT CCT-3′) as described previously [11]. We expressed and purified recombinant Pv50 lacking the signal peptide (ΔSP) by cell-free wheat germ expression [12]. Briefly, the DNA fragment was amplified, cloned into the pDNA vector pEU-E01-His-TEV-MCS (CellFree Sciences, Matsuyama, Japan), and used for recombinant protein expression. The wheat germ cell-free expression system was applied for recombinant Pv50 protein expression and purified using a Ni-Sepharose column as described previously [14].

### Immunization of mice and rabbits with Pv50

Female BALB/c mice (DBL Co., Seoul, ROK) were used at 6–8 weeks of age. Groups of 3 mice were injected intraperitoneally with approximately 20 µg of Pv50 and phosphate-buffered saline (PBS) with Freund’s complete adjuvant, respectively (Sigma-Aldrich, St. Louis, MO, USA). Using the same amount of antigen with Freund’s incomplete adjuvant (Sigma-Aldrich), booster injections were administered 3 and 6 weeks later. Mouse blood samples were collected 2 weeks after the each booster.

To generate antibodies against Pv50 for immunofluorescence analysis, 250 μg of purified recombinant Pv50 protein was used for immunization with Freund’s complete adjuvant in one Japanese white rabbit administered by subcutaneous injection 3 times at 3-week intervals. After the last booster injection, antisera were collected on day 14.

### SDS-PAGE and western blot analysis

The recombinant Pv50 and *P. vivax* schizont-stage parasite lysates were separated by 12% SDS-PAGE and then stained with Coomassie brilliant blue. For western blot analysis, recombinant proteins were electrophoretically transferred to polyvinylfluoride membranes (Millipore Corp., Billerica, MA, USA), and incubated with blocking buffer (5% non-fat milk in PBS containing 0.2% Tween 20, PBS/T) for 1 h at 37 °C. After blocking, penta anti-His antibody, mouse immune sera, rabbit immune sera, or mixed patient serum were diluted by 200-fold with PBS/T, and the specific quality of the His-tagged recombinant protein and immune serum was examined using the secondary antibody IRDye^®^ goat anti-mouse (1:10,000 dilution), IRDye^®^ goat anti-rabbit (1:20,000 dilution), or IRDye^®^ goat anti-human (1:20,000) (LI-COR Biosciences, Lincoln, NE, USA). The fluorescence signals from the reaction were scanned on an Odyssey infrared imaging system (LI-COR Biosciences) and analyzed with Odyssey software (Li-Cor Biosciences).

### Indirect immunofluorescence assay (IFA)

The enriched schizont-stage parasites were purified by Percoll gradient centrifugation, spotted onto a multi-well slide, fixed in ice-cold acetone for 3 min, air-dried, and stored at -80 °C. Before use, the slides were thawed on silica gel blue (Samchun Chemical, Pyeongtaek, Gyeonggi, ROK) and blocked with PBS containing 5% non-fat milk at 37 °C for 30 min. Next, the slides were incubated with 1:200 diluted primary antibodies, mouse anti-MSP1-19 [15] and rabbit anti-Pv50, at 37 °C for 1 h. After the reaction, the slides were stained with Alexa 546-conjugated goat anti-rabbit IgG secondary antibody (Ab) or Alexa 488-conjugated goat anti-mouse IgG secondary Ab (Invitrogen, Carlsbad, CA, USA) and nuclei were stained with 4′,6-diamidino-2-phenylindole (DAPI; Invitrogen) at 37 °C for 30 min. The slides were mounted with cover slips using mounting medium ProLong Gold antifade reagent (Invitrogen) and observed under oil immersion in a confocal laser scanning FV200 microscope (Olympus, Tokyo, Japan) equipped with 20× dry and 60× oil objectives. Images were visualized with FV10-ASW v.3.0 viewer software, the overlap coefficient analyzed with Image J (Softonic International, Barcelona, Barcelona, SA), and adjusted for publication with Adobe Photoshop CS5 (Adobe Systems, San Jose, CA, USA).

### Protein arrays

In the present study, we prepared amine-coated slides as described previously [11, 12]. Briefly, 112 cases of vivax malaria and 80 unexposed individual serum samples were used for humoral immune response analysis. Purified Pv50 protein was spotted into duplicate wells of the arrays at 100 ng/μl in PBS and incubated for 1 h at 37 °C. After blocking with 1.0 μl of blocking buffer (5% bovine serum albumin in PBS with 0.1% Tween 20, PBS/T) for 1 h at 37 °C, the chips were probed with initial pre-absorbed sera from malaria patient or healthy individual sera (1:10 dilution) in wheat germ lysate (1:100 dilution) to block the anti-wheat germ antibodies. The native Pv50 protein was detected by Alexa Fluor 546 goat anti-human IgG (10 ng/μl; Invitrogen) in PBS/T, and scanned in a fluorescence scanner (ScanArray Express; PerkinElmer, Waltham, MA, USA) [11]. The cut-off value was equal to the mean plus two standard deviations (SD) of the mean intensity of 80 negative samples.

### Enzyme-linked immunosorbent assay (ELISA)

We analyzed further IgG subclass from Pv50 immune mouse sera. Briefly, the recombinant Pv50 (5 µg/ml) was coated for testing immune mouse sera titers. One hundred microliters of the purified mouse IgG1, IgG2a, IgG2b (Invitrogen, Carlsbad, CA, USA) and IgG3 (BD Pharmingen, San Diego, CA, USA) were each coated on 96-well plates at concentrations of 256, 128, 64, 32, 16, 8 and 4 ng/ml, respectively. The coated proteins were incubated with immune mouse sera in a 1:1000 dilution with PBS-T. The reaction was detected by anti-mouse IgG1, IgG2a, IgG2b and IgG3 antibodies conjugated HRP (Invitrogen) at dilutions of 1:1000, 1:1000, 1:2000 and 1:1000, respectively. Color intensity was measured and calculated using a log-log curve fit.

### Splenocyte proliferation and cytokine production

Two weeks after the third immunization, the spleens were removed from mice as previously described [15]. Briefly, splenocytes from Pv-50 immunized mice and PBS immunized mice were resuspended at concentrations of 2.5 × 10^6^ cells/ml in complete RPMI1640 (Gibco Life Technologies, Grand Island, NY, USA) in 96-well culture plates. Pv50 (200 µl) at a concentration of 10 µg/ml was mixed with the cells. Additionally, splenocytes were stimulated with 5 µg/ml of concanavalin A (Con A; Sigma-Aldrich) or 10 µg/ml of lipopolysaccharides (LPS; Sigma-Aldrich) as a positive control, while medium mixed with cells without any other fraction was used as a negative control. After 48 h of culture, 100 µl of supernatant/well was collected and stored at -70 °C for cytokine analysis. Enhanced cell viability buffer (50 µl; Daeil Lab Service Co. Ltd., Seoul, ROK) was added to 50 µl of splenocyte culture medium for the last 4 h of a 48-h incubation period. The optical density at 450 nm of each well was analyzed using an ELISA plate reader. The stimulation index (SI) was calculated as follows: mean OD_450_ of triplicate test wells/mean OD_450_ + two standard deviations of sextuplicate control wells. Proliferation was considered as positive when the SI was > 1.

Cytokine concentrations were measured in culture supernatants from immunized mice using a BD™ CBA Flex Set kit (BD Biosciences, San Diego, CA, USA). The cytokines assayed included mouse gamma interferon (IFN-γ), tumor necrosis factor (TNF), interleukin-12p70 (IL-12p70), IL-2, IL-4, IL-5 and IL-10. The results were acquired on a FACSAria™ II Cell sorter (BD Biosciences) according to the manufacturer’s instructions and analyzed using FCAP array software (Soft Flow, Kedves, Hungary).

### *In situ* proximity ligation assay (PLA)

The interaction between Pv50 and PvMSP1 proteins in *P. vivax* parasites detected by *in situ* PLA assay using a Duolink In Situ kit (Olink Bioscience, Uppsala, Sweden). Slides smeared with parasite-infected blood samples were fixed with ice-cold acetone. These slides were blocked with PBS-T containing 5% non-fat milk at 37 °C for 60 min. For primary antibody reactions, the slides were double-labeled at 37 °C for 1 h with the following antibodies: rabbit anti-Pv50 (1:200 dilution) and mouse anti-PvMSP1-42 (1:100 dilution), rabbit anti-Pv50 (1:200 dilution) and mouse anti-PvMSP10 (1:100 dilution). The primary antibody solution was taped off from the slides, which were then washed in PBS-T. The slides were incubated with anti-mouse MINUS, anti-rabbit PLUS or PLA probes mixture in a humidity chamber for 1 h at 37 °C. After washing, the slides were incubated with ligation mixture in a humidity chamber for 30 min at 37 °C. The slides were washed as above and incubated with amplification-polymerase solution in a humidity chamber for 1.5 h at 37 °C. After washing, the slides were mounted with a cover slip using a minimal volume of mounting medium with DAPI. All images were visualized and analyzed as IFA.

### Determination of dissociation constants (*Kd*)

Pv50 (100 μl, 100 μg/ml) in 100 mM sodium bicarbonate buffer (pH 8.3) mixed with 2 μl of 1 mg/ml Cy5 mono NHS-ester (Amersham Bioscience, Piscataway, NJ, USA) in 10% dimethyl sulfoxide (Sigma-Aldrich) was incubated for 2 h on ice. To quench the reactions, 5 μl of 1 M Tris-HCl (pH 8.0) was added to the reaction solution. Cy5-conjugated TG2 was eluted by centrifugation for 3 min at 1050×*g*. To calculate the molar concentrations of Cy5 and Pv50 in Cy5-conjugated Pv50, the molar extinction coefficients of pure Pv50 at 280 nm and Cy5 at 280 and 650 nm were determined using a Nanodrop^®^ ND-1000 UV-Vis spectrophotometer (NanoDrop Technologies, Wilmington, DE, USA) [16]. The Pv50-related proteins were also labeled with Cy5 NHS ester and the absorbance of Cy5-conjugates was determined using the Nanodrop^®^ ND-1000 UV-Vis spectrophotometer as described above. Protein concentrations of Cy5-conjugates were determined using the same equation. To detect interactions of Pv50 with other *P. vivax* merozoite surface proteins, we used 5 known proteins or fragments. Five proteins at gradient concentrations were immobilized onto well-type amine chip arrays for 1 h at 37 °C and blocked with blocking buffer [2 μM bovine serum albumin containing 0.1% (v/v) Tween 20 in PBS] for 30 min at 37 °C, and then washed with 0.1% Tween 20 in PBS and milli-Q-purified water. Cy5-conjugated Pv50 (20 μg/ml) dissolved in 30 μl of a solution containing 40 mM Tris-HCl (pH 7.5), 140 mM NaCl, 2 mM CaCl_2_, 50 mM dithiothreitol, 5 mM 5-(biotinamido) pentylamine (BAPA, Pierce, Rockford, IL, USA) and 0.01% (v/v) Triton X-100 was applied to each well and incubated at 37 °C for 30 min. After washing with 0.1% (v/v) Tween-20 in PBS and deionized water, BAPA incorporation catalyzed by Pv50 was probed with 10 μg/ml of Cy3-conjugated streptavidin (Sigma-Aldrich) at 37 °C for 30 min. The arrays were dried under compressed air after washing and the chips were scanned with a fluorescence scanner using 543- and 633-nm lasers (PerkinElmer). Fluorescence intensities of the chip array were analyzed using the ScanArray Express program (PerkinElmer). Fluorescence intensities at the Cy3 and Cy5 channels represent Pv50 activities and interactions, respectively, with merozoite surface proteins. We quantitatively analyzed the binding affinities of Pv50 with 5 merozoite surface proteins by determining the dissociation constants (*Kd*).

By using GraphPad PRISM (GraphPad, Inc., La Jolla, CA, USA), a modified Langmuir isotherm formulation was used to calculate the *Kd* values:

$$Fobs = \frac{{F_{{\max} } \times \left( {protein} \right)}}{{Kd + \left( {protein} \right)}} + background$$

*F*_*obs*_ is equivalent to fluorescence intensity of triplicate spots, *F*_*max*_ is the signal expected from maximum fluorescence at complete saturation, (protein) is the protein concentration on the surface, and the *Kd* can be computed graphically at equilibrium.

### Statistical analysis

Data were analyzed using GraphPad Prism and SigmaPlot software (Systat Software, Inc., San Jose, CA, USA). The Mann-Whitney test was used to compare differences between the means of each group for statistical significance. *P* < 0.05 was considered to indicate a significant difference. Simple scatter-regression was used to generate a standard curve.

## Results

### Description of PVX_087140 orthologs in *Plasmodium* spp.

**Fig. 1:**
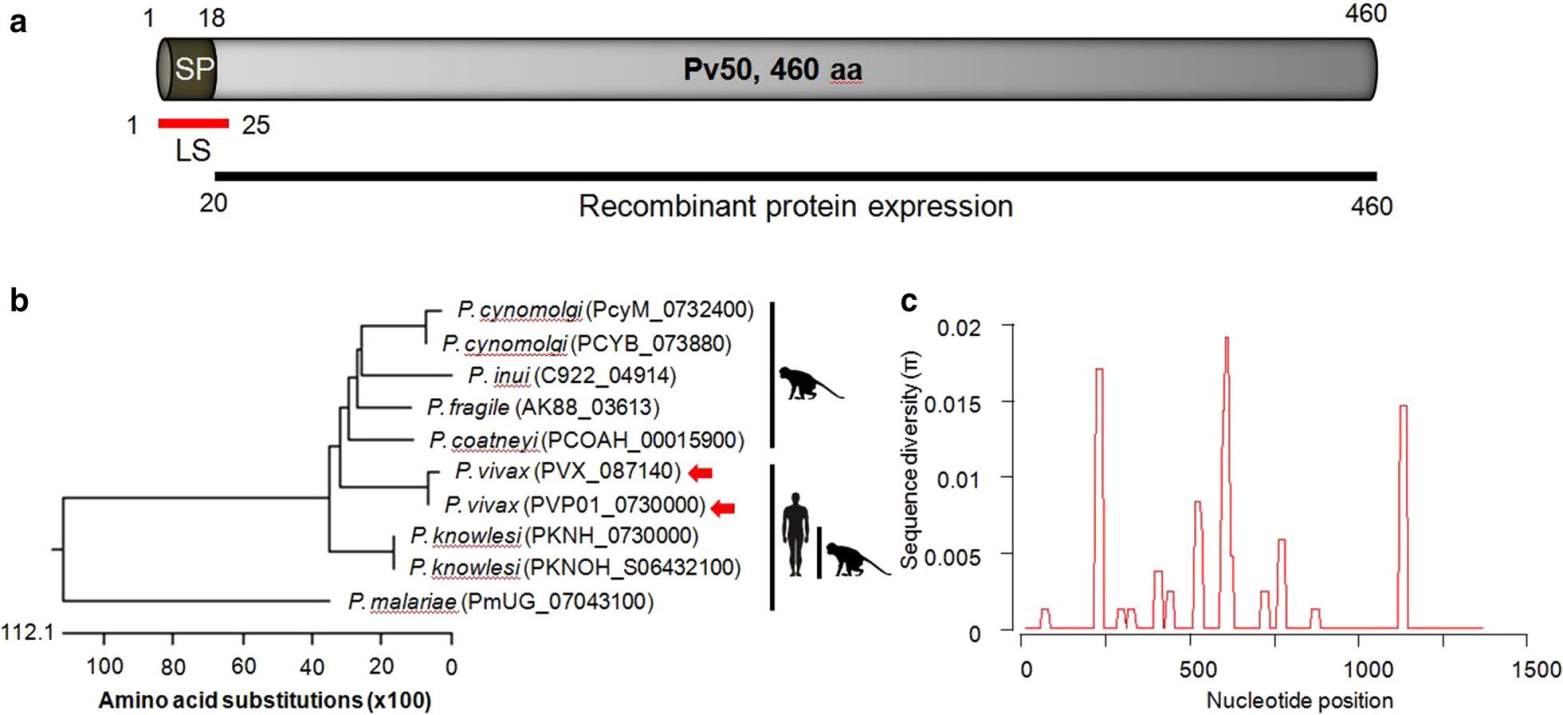
Primary structure and sequence characterization of Pv50. **a** Schematic diagram of Pv50. The Pv50 protein contains 460 amino acids, with a calculated molecular mass of 50.4 kDa. Indicated are the signal peptide (amino acid (aa) positions 1–19) and lipid attachment site (aa, 1–25). Truncated Pv50 (20–460 aa) was constructed for expression. **b** Phylogenetic tree showing the relationships among protein sequences of *P. cynomolgi*, *P. inui*, *P. fragile*, *P. coatneyi*, *P. vivax*, *P. knowlesi* and *P. malariae 50* genes. The position of *P. vivax pv50* is indicated with a red arrow. **c** Sliding window plot showing the nucleotide diversity of the *pv50* in 64 worldwide isolate sequences with window size of 25 and a step size of 5

*Pv50* consisted of 464 residues from the Sal-I strain including a signal peptide sequence comprised of the first 18 amino acids and a lipid attachment domain comprised of the first 25 amino acids (Fig. 1a). Orthologues of *pv50* in primate malaria species *P. cynomolgi* (Strain M, PcyM_0732400; Strain B, PCYB_073880), *P. inui* (San Antonio 1, C922_04914), *P. fragile* (Strain nilgiri, AK88_03613), *P. coatneyi* (Hackeri, PCOAH_00015900) and human invasive malaria species *P. vivax* (Sal-I, PVX_087140; P01, PVP01_0730000), *P. knowlesi* (Strain H, PKNH_07300000; Malayan Strain Pk1 A, PKNOH_S06432100) and *P. malariae* (UG01, PmUG_07043100) showed similar structures in the C- and N-terminal regions with highly conserved cysteine residues (Additional file 1: Figure S1). The cluster results were used to divide the samples into the old monkey invasive malaria group and human invasive malaria group; however, *P. malariae* was distant from the other orthologs of *pv50* (Fig. 1b).

Sequence diversity of *pv50* within 64 isolates was found in 17 segregating sites among 1383 nucleotide sites showing a sequence diversity (π) of 0.00142, indicating that *pv50* contains few polymorphisms (Fig. 1c). The most variable amino acid site was position 78, with 18 isolates changed from Thr to Pro among the 64 isolates (Additional file 2: Table S1). Additionally, amino acid changes occurred simultaneously at H203D, Y204H and D207G within four isolates (Additional file 2: Table S1).

### Expression, purification and western blotting analysis of Pv50 with immune serum samples

**Fig. 2:**
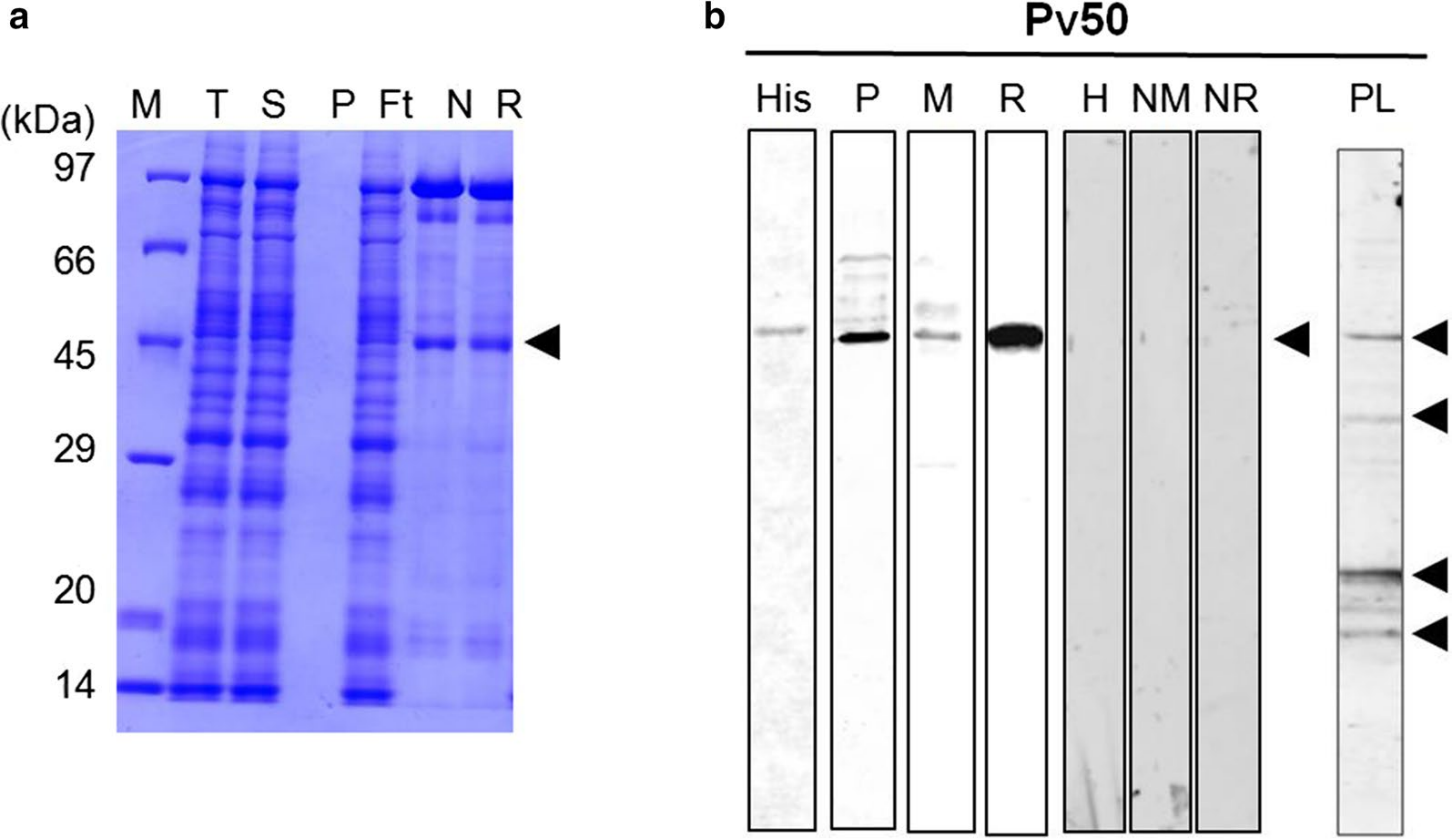
Recombinant Pv50 protein for expression. **a** The purification progress of Pv50 (46 kDa) was evaluated by 12.5% SDS-PAGE. **b** Western blot analysis of recombinant Pv50 with penta anti-His antibody (His), mouse immune sera (M), mixed vivax patient sera (P), rabbit immune sera (R), and schizont-stage parasite lysates (PL) under reducing conditions probed with IgG antibody from Pv50 rabbit immune sera. Arrowheads indicate specific bands for each recombinant protein. *Abbreviations*: M, protein marker; T, total translation mix; S: supernatant; P, purification; Ft: flow through; N, elution under non-reducing; R, elution under reducing

To characterize this putative high antigenic protein, we expressed Pv50 using a cell-free expression system with constructs based on the Sal-I strain gene sequence. The expression vectors encoding the truncated Pv50 (ΔSP) were prepared and the protein was successfully purified under non-denaturing conditions as shown in Fig. 2a. Recombinant Pv50 migrated as a single band of ~ 46 kDa in SDS-PAGE, which was slightly smaller than the predicted size.

To analyze the localization of Pv50, polyclonal antibodies were produced against the protein in a rabbit with recombinant Pv50. To assess Pv50 immunogenicity, we immunized mice with this recombinant Pv50. The corresponding immunoblots (Fig. 2b) probed with an anti-His tag monoclonal antibody (His), mixture of vivax patient sera (P), anti-Pv50 mouse immune serum (M) and anti-Pv50 rabbit immune serum (R) revealed similar and strong patterns of migration for Pv50. Pre-immune mouse sera (NM), rabbit sera (NR) and malaria naïve human serum (H) samples obtained from individuals living in non-malaria endemic areas were used as negative controls, and no reaction was observed in this negative control group.

#### Humoral immune response analysis of Pv50

**Fig. 3:**
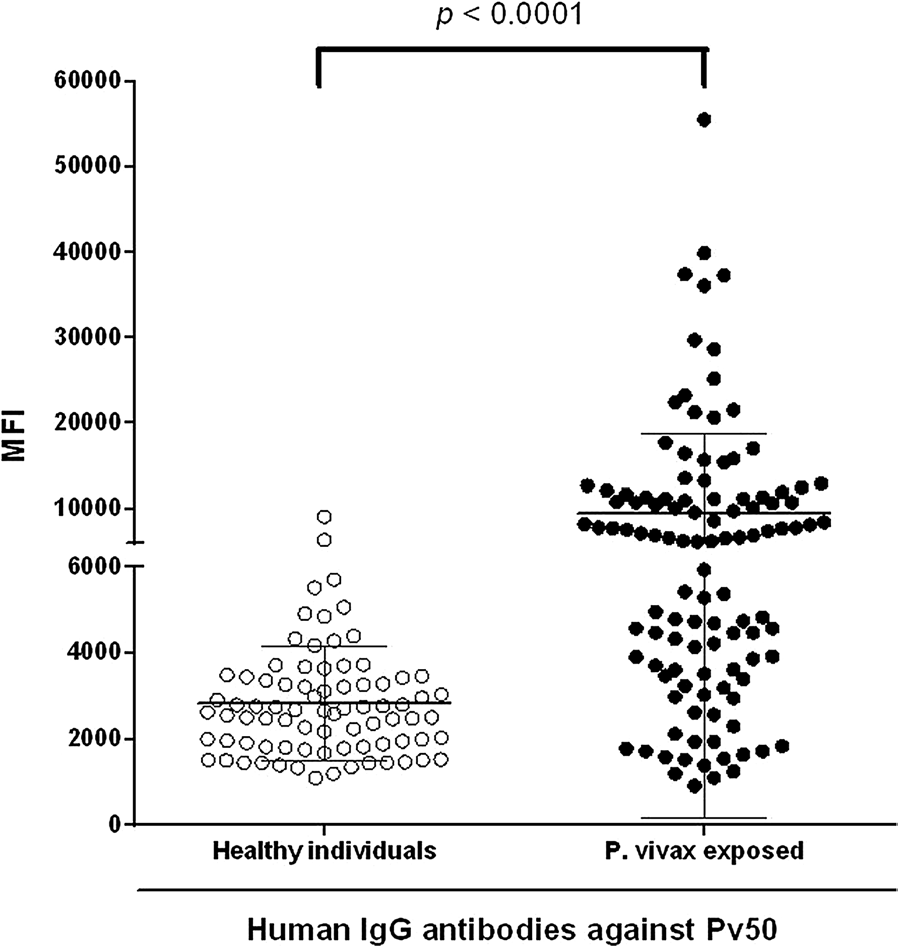
IgG antibody responses to Pv50 using protein microarrays. Recombinant Pv50 from the sera of malaria patients (positive) and healthy individual samples (negative) from the ROK was probed. Significant differences in total IgG prevalence were observed with high specificity between patients with vivax and healthy individuals (*P* < 0.0001). The *P-*values were calculated using a Mann-Whitney test. The bar indicates the mean plus ± SD

To further evaluate humoral immune responses against purified Pv50, we screened for the presence of antibodies in human sera with purified Pv50 protein by protein array. Antibody responses against Pv50 were determined from 112 patients with *P. vivax* and 80 serum samples from healthy individuals. *P. vivax*-exposed individual sera samples showed a significantly higher MFI of total IgG than those from malaria-naïve subjects (Fig. 3, *χ*^2^ = 19.47, *df* = 1, *P* < 0.0001). The prevalence of anti-Pv50 antibody showed a sensitivity of 42.9% (48 in 112 vivax samples > cut-off value 5480) and specificity of 93.8% (75 in 80 healthy samples < 5480) (Table 1). These data confirm the high immunogenicity of Pv50, in terms of the results from a previous study [11], and suggest the considerable immunogenicity of Pv50. This may be because *pv50* contains few polymorphisms [17] and the parasite surface protein is exposed to the host immune system.

**Table 1:** Prevalence (% positive), 95% confidence intervals and mean fluorescence of intensity of IgG responses to *Plasmodium vivax* 50 in human patients and healthy individual serum samples

Antigen	No. of patient samples			MFI	95% CI (%)	No. of healthy samples			MFI	95% CI (%)	*P*-value^c^
	Positive	Negative	Total (%)^a^			Positive	Negative	Total (%)^b^			
Pv50	48	64	112 (42.9)	9501	41.2–60.8	5	75	80 (6.2)	2830	91.2–98.9	<0.0001

^a^Sensitivity: % positive in patient samples

^b^Specificity: % negative in healthy samples

^c^Differences in the total IgG prevalence for each antigen between vivax patients and healthy individuals were calculated with the Mann-Whitney test; *P *< 0.05 was considered as statistically significant

*Abbreviations*: MFI, mean fluorescence intensity; CI, confidence interval

#### Pv50 localizes on the merozoite surface

**Fig. 4:**
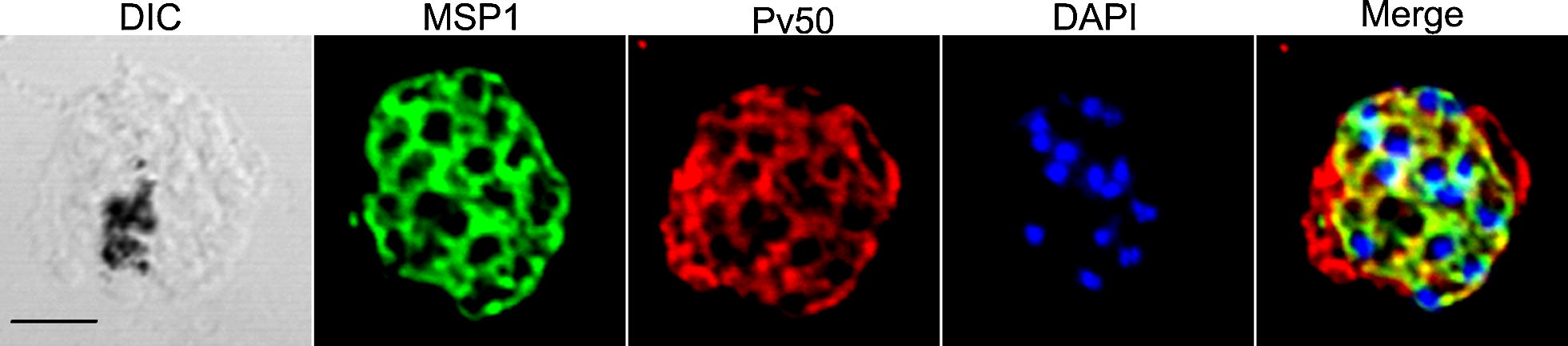
Localization of Pv50 mature schizont stage. Schizont-stage parasites were dual-labeled with antisera against Pv50 (red color), PvMSP1-19 (merozoite surface marker, green color), and nuclei were stained with DAPI (blue). *Scale-bar*: 2.5 μm

To determine the localization of the native Pv50 protein in wild-type mature schizonts, an immunofluorescence assay was carried out using anti-Pv50 and anti-PvMSP1-19 antibodies. In blood-stage vivax parasites, Pv50 was localized to the outer circle of each merozoite (Fig. 4, red color). Because Pv50 is only highly expressed in the schizont stage during the 48-h intraerythrocytic cycle [13], we compared the localization of Pv50 with that of the merozoite surface protein PvMSP1 only in the schizont stage (Fig. 4, green color). The data showed that the Pv50 signal was almost merged with that of MSP1 (overlap coefficient 0.99). Thus, Pv50 is a merozoite surface protein.

### Cytophilic antibodies were major components against Pv50 in immunized mice

**Fig. 5:**
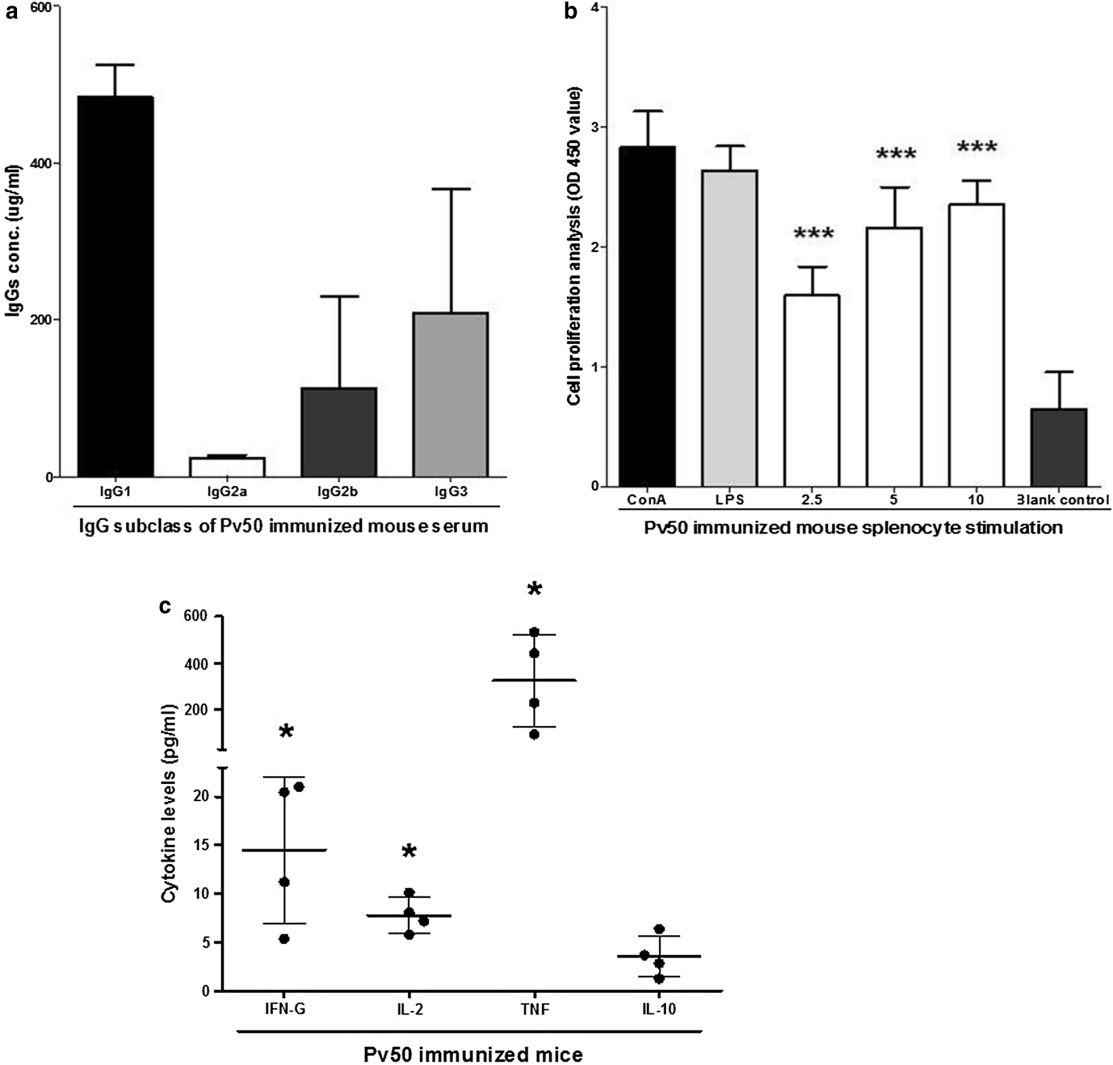
Immunogenicity analysis of Pv50. OD_450_ value of culture medium with recombinant Pv50 (2.5, 5 and 10 µg/ml), ConA, LPS, or only culture medium as control. **a** Proliferation of splenocytes is shown after stimulation with Con A or LPS as a positive control. The results are expressed as the mean value of OD_450_ ± SD. **b** Cytokine levels from 72 h cultured supernatants of splenocytes from Pv50-immunized BALB/c mice stimulated *in vitro* with Pv50. The black horizontal bars represent the cytokine geometric means ± 3SD. **c** Specific IgG1 (black), IgG2b (dark gray), IgG3 (light gray) and IgG2a (white) against the immunogen itself were determined by ELISA. The results are expressed as the mean titers ± SD

The immunogenicity induced in patients with *P. vivax* against Pv50 can be reflected in the specific immunogenicity induced by Pv50 in immunized mice. Here, after three immunizations with Pv50, the IgG1, IgG2a, IgG2b and IgG3 concentrations in the sera of mice immunized with Pv50 were 483.6 (450.9–530.2), 22.4 (18.4–27.6), 112.9 (39.8–247.8), and 208.6 (86.1–387.3) µg/ml, respectively (Fig. 5a). The non-cytophilic antibodies IgG1 and IgG3 were key components in the antibody response in Pv50-immunized mice.

### Effect of immunogen components on the Th1/Th2 balance

After stimulation with recombinant Pv50 antigen both *in vivo* and *in vitro*, we found that the splenocyte population in mice increased as the Pv50 antigen concentration increased (Fig. 5b, *P* < 0.0001). To determine the Th1/Th2 balance in T-cell responses, the levels of TNF, IFN-γ, IL-2, IL-4, IL-5, IL-10 and IL-12p70 cytokines were determined in the culture supernatants at 48 h after *in vitro* stimulation with the four immunogens. The levels of IL-2 (7.8; 5.8–10.1 pg/ml, *P* = 0.02), IFN-γ (14.5; 5.4–21.0 pg/ml, *P* = 0.03), and TNF (322.2; 90.0–532.2 pg/ml, *P* = 0.02) were significantly higher than that of IL-10 (3.5; 1.3–6.3 pg/ml) (Fig. 5c). The results obtained in splenocytes of Pv50-immunized mice indicate a Th1 response, with TNF, IFN-γ and IL-2 as the predominantly secreted cytokines. The Th2 profile was predominated by IL-10, IL-4 and IL-5, and only IL-10 was able to be detected in this case. Splenocytes from mice immunized with PBS were also stimulated with Pv50, but the cytokine levels generated were borderline-negative.

#### Determination of Pv50 affinity with merozoite surface proteins

**Fig. 6:**
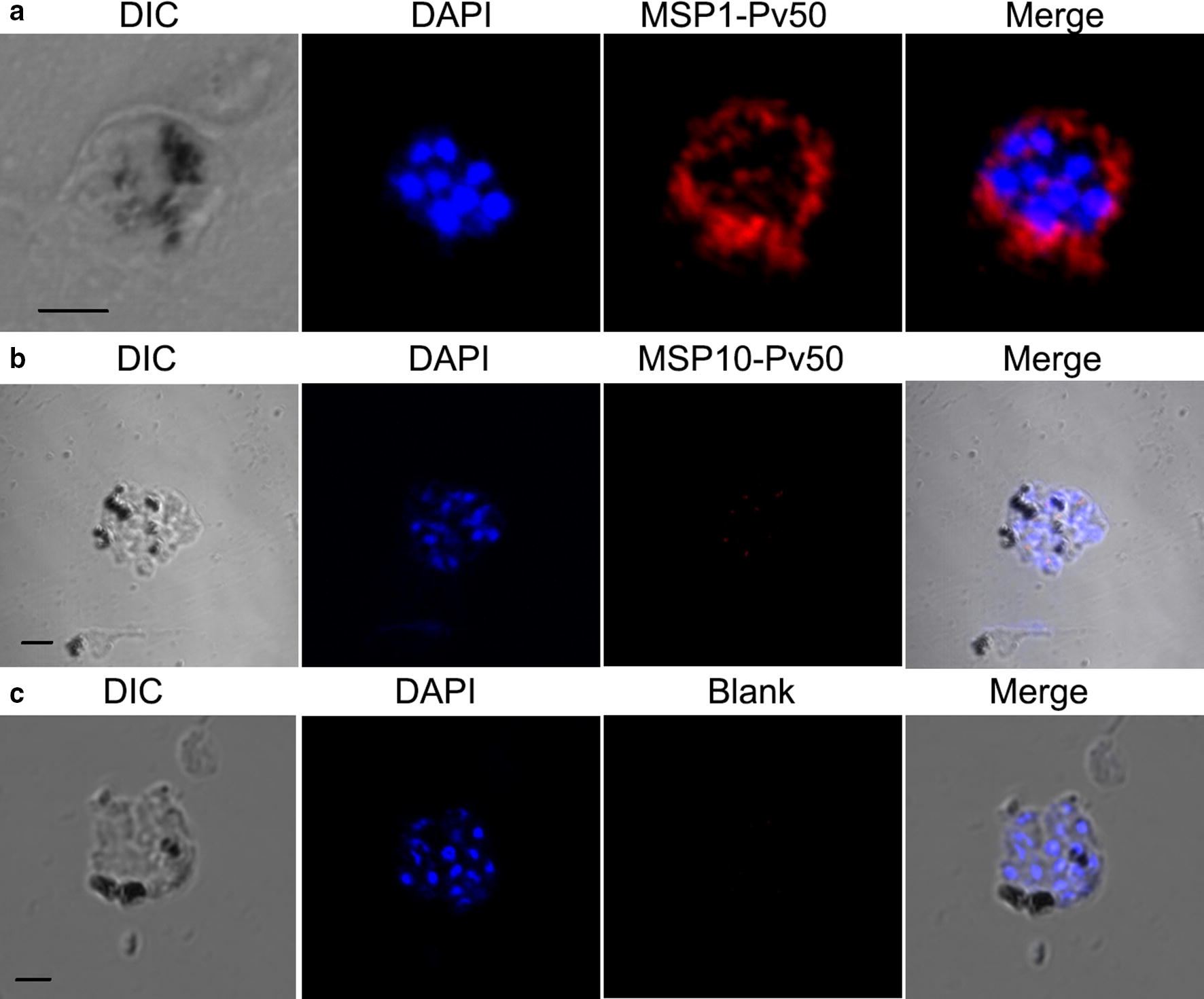
The interaction between Pv50 and PvMSP1. The interaction were visualized by probing mouse and rabbit immune sera, and staining parasites with probes termed anti-Mouse MINUS and anti-rabbit PLUS, the hybridization probes were labeled with Texas Red (Red), and nuclei were stained with DAPI (blue). **a** PvMSP1 interacted with Pv50 in the schizont stage. **b** PvMSP10 did not interact with Pv50 in the schizont stage. **c** PLA assay developed without first antibody as blank control. *Scale-bar*: 2.5 μm

**Fig. 7:**
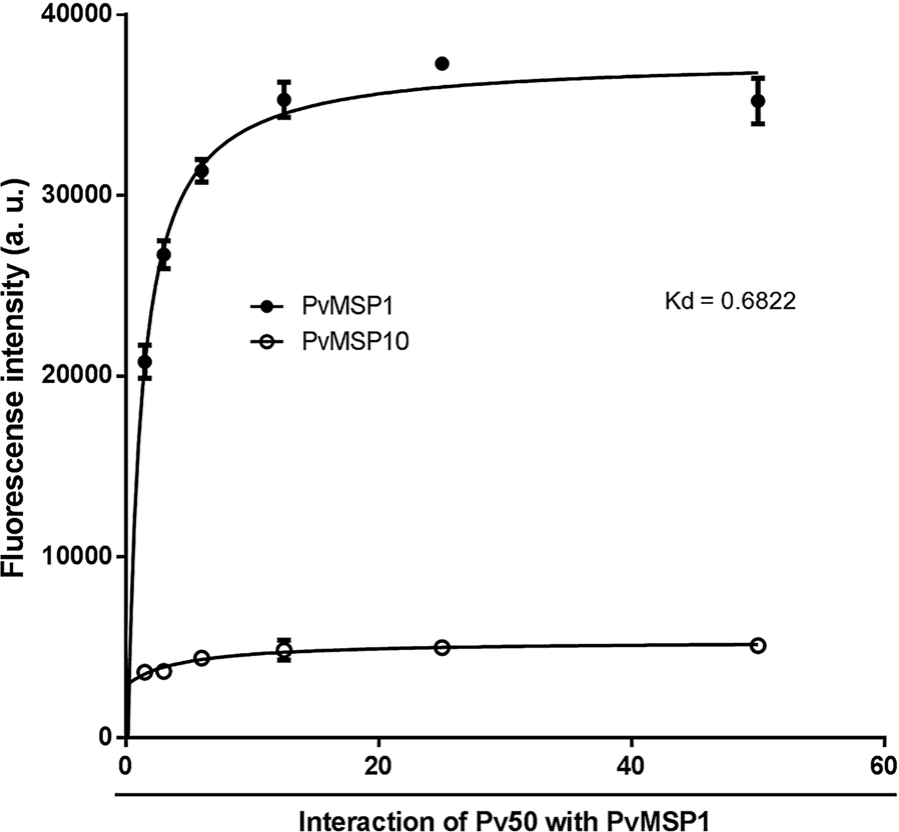
Kinetics for interaction between Pv50 and *P. vivax* MSP1. Reaction mixtures (1 μl) containing 10 μg/ml Cy5-conjugated Pv50 were applied to protein arrays for 30 min. Interactions of Pv50 with PvMSP1 were determined as described in methods. The results are expressed as the means of fluorescence intensities ± SD from three separate experiments

To investigate possible interactions between Pv50 and merozoite surface proteins in native *P*. *vivax* parasites *in vivo*, we applied the PLA assay using mouse or rabbit immune sera as primary antibodies. We observed strong fluorescence signals in *P*. *vivax* parasites (Fig. 6a), which suggested that Pv50 might interact with PvMSP1 in schizont. A comparatively faint signal intensity between PvMSP10 and Pv50 (Fig. 6b) was observed. The blank control has been developed that *P. vivax* parasites only incubated with second antibody (Fig. 6c). The signals detected (as shown in Fig. 6a) were specific, suggesting that Pv50 might interact with PvMSP1 in *P*. *vivax* parasites. To quantitatively analyze the interaction of Pv50 with merozoite surface protein(s), fluorescence intensities observed using the Cy5 channel from the simultaneous activity and binding assay of Pv50 were plotted against the concentrations of 5 merozoite surface proteins and fitted using the equation. As expected, only the PvMSP1 binding affinity (*Kd*) was determined and ranged from 1.5 to 50.0 nM but was not detectable for other proteins (Fig. 7). Three groups were observed based on the log *Kd* values (log*Kd*): strong (log*Kd* < 1, IS), weak (log*Kd* > 1, IW) and nondetectable (IN) interaction groups. PvMSP1 showed a strong interaction with Pv50 (log*Kd* < 1). The *Kd* value for PvMSP10 was undetectable, indicating that PvMSP10 did not interact with Pv50.

## Discussion

Pv50, a conserved blood stage protein, is strongly recognized by antibodies from individuals naturally infected with *P. vivax*, confirming its potential as a vaccine candidate. The interaction between Pv50 and PvMSP1 may provide a rational strategy for PvMSP1-based vaccine design.

Once infected, red blood cells rupture and merozoite surface proteins are exposed to the host immune system, and thus can be recognized by immune sera from injected patients from endemic areas [9, 18, 19]. These antibodies play an essential role in protection against blood-stage malaria parasites [20, 21]. In the present study, Pv50 was shown to be localized to the merozoite surface (Fig. 4). To characterize the antibody responses induced by Pv50, we determined the IgG antibody prevalence in 112 patients with *P*. *vivax* mono-infection from an endemic area in the ROK and the results showed that nearly 42.9% of this population had antibodies against Pv50. These data confirm the high immunogenicity of Pv50 as described previously [11] and indicate the considerable immunogenicity of Pv50. This may be because of the low number of polymorphisms in *pv50* [17] and that the parasite surface protein is exposed to the host immune system.

IgG subclass (IgG1, IgG2a, IgG2b, and IgG3) antibodies in immunized mice strongly recognized Pv50 (Fig. 5c), and non-cytophilic IgG subclasses (IgG1 and IgG3) play a predominant role in immune response in Pv50-immunized mice. That was different from previous findings that cytophilic antibodies IgG2a were the dominant antimalarial isotype in rodent malaria [22]. Additionally, Th1 predominantly induced by Pv50 *in vitro* was detected (Fig. 4b), and Th1 responses are important for the clearance of malaria parasites [21, 23].

The merozoite surface localization of Pv50 may initiate the invasion cascade. As for MSP6 or MSP7, there is no GPI-anchor motif in Pv50, indicating that Pv50 anchors into the merozoite surface membrane with the help of other merozoite surface GPI-anchor proteins. MSP1 is the most abundant protein anchored into this membrane and plays an essential role in merozoite invasion of red blood cells [24]. PvMSP1 is a malaria vaccine candidate because of its immunogenic effect in a large proportion of individuals exposed to malaria [25, 26]. A key challenge in developing an effective malaria vaccine that can block erythrocyte invasion is determining the molecular interaction(s) among parasite surface proteins as well as with host cell encoded receptors. PfMSP1 was shown to be associated with other proteins (MSP3, MSP6 and MSP7) to form a complex [8, 27]. Multiple *P. falciparum* MSP1 complexes mediate binding to human red blood cells [28]. However, no studies have shown that PvMSP1 interact with other proteins that may limit the development of effective PvMSP1-based vaccines. In this study, PvMSP1 was identified as a *P. vivax* merozoite surface protein that strongly interacts with Pv50. Further studies aimed at assessing its immunogenicity and protection-inducing ability in the *Aotus* monkey model are thus recommended.

## Conclusions

In conclusion, based on immune profiling information and the specific interaction with PvMSP1, Pv50 may be useful for developing a PvMSP1-based subunit vaccine.

## Additional files

**Additional file 1: Figure S1.** Pv50 amino acid sequence alignment with orthologues.

**Additional file 2: Table S1.** *Pv50* gene sequence diversity.

## Abbreviations

Pv50: *P. vivax* 50-kDa protein
GPI: glycosylphosphatidylinositol
π: sequence diversity
PBS: phosphate-buffed saline
Con A: concanavalin A
LPS: lipopolysaccharides
SI: stimulation index
IFN-γ: gamma interferon
TNF: tumor necrosis factor
IL: interleukin
*Kd*: dissociation constant
His: anti-His tag monoclonal antibody
P: mixture of vivax patient sera
M: anti-Pv50 mouse immune serum
R: anti-Pv50 rabbit immune serum
NM: pre-immune mouse sera
NR: rabbit sera
H: malaria naïve human serum
IN: non-detectable
