# Supplementary material for: Identification and characterization of Pv50, a novel Plasmodium vivax merozoite surface protein
Source: Parasit Vectors. 2019 Apr 18;12:176. doi: 10.1186/s13071-019-3434-7 (PMC6474066; doi:10.1186/s13071-019-3434-7)
Supplement: Supplementary file 1 — Additional file 1: Figure S1. Pv50 amino acid sequence alignment with orthologues. [file 13071_2019_3434_MOESM1_ESM.docx]

**Additional file 1: Figure S1 Pv50 amino acid sequence alignment with orthologues.**

*P. vivax* (PVX_087140) MKLTCTLFFLLAVASNLGSNFFTSCSVDSSSVSQQTYRVPYLQRLSELIALSSHQGLSDKRADEKKNLRRKREDSDSTQV 80

*P. vivax* (PVP01_0730000) MKLTCTLFFLLAVASNLGSNFFTSCSVDSSSVSQQTYRVPYLQRLSELIALSSHQGLSDKRADEKKNLRRKREDSDSTQV 80

*P. malariae* (PmUG_07043100) MKLALGLFLTLAIASNFGSRFFGYLAMGDLLVSGQMNNSPYTMTLSKMNTKITNNELNDKQEEQNKNLRRKRQD----DS 76

*P. knowlesi* (PKNH_0730000) MKLACALFFLLAVASNCGSHFFTFCSVDNSSVSQQTHRIPYLQRLSELIALSSYRELSDKRAEEKKNLRRKREDSYTPHI 80

*P. knowlesi* (PKNOH_S06432100) MKLACALFFLLAVASNCGSHFFTFCSVDNSSVSQQTHRIPYLQRLSELIALSSYRELSDKRAEEKKNLRRKREDSYTPHI 80

*P. cynomolgi* (PcyM_0732400) MKLTCALFVLLAVASNWGSHFFTSCSVDNSSVSQQTYRAPYLQRLSELIALSSYQELSDKRAEEKKNLRRKREDSDSPHV 80

*P. cynomolgi* (PCYB_073880) MKLTCALFVLLAVAT----------------------------------------------------------------- 15

*P. coatneyi* (PCOAH_00015900) MKLACALFLLLAVASNWGSHFFASCSVDNSSVSQQTHRVPYLQRLSELIALSSYQGLSDKREEEKKNLRRKREDSDSPQV 80

*P. fragile* (AK88_03613) MKLACALFLVLAVASNWGSHFFTSCFVDNSSLSQQTYRVPYLQRLSELIALSPYQELSDKRAEEKKNLRRKREDNDSPNV 80

*P. inui* (C922_04914) MKLTCALFFLLAVASNWGPHFFTSCSVDNSSVSEQTYRVPYLQRLSELIALSSHEELSDKRAEEKNNLRKKREDNDSPHT 80

*P. vivax* (PVX_087140) ADD-----EDVTSAATHVEEQA---EDAEEDLTSNKAGIPQEEDASGEGISLDGEQWEDEDEHDGGADPSGPTPDYHYGE 152

*P. vivax* (PVP01_0730000) ADD-----EDVTSAATHVEEQA---EDAEEDLTSNKAGIPQEEDASGEGISLDGEQWEDEDEHDGGADPSGPTPDYHYGE 152

*P. malariae* (PmUG_07043100) SDDVSSSDSDIPFTS------------------------------NGDDKTIDDQVSYEDSDGNAAPGNAAP-------- 118

*P. knowlesi* (PKNH_0730000) ADDSSVNEEEVTIATINGEEKVKKTEKAEEDIESDYVGVPKEEDDSGKGISLDEEAGSIDYEHSMNAEAAGSIDYEHSVN 160

*P. knowlesi* (PKNOH_S06432100) ADDSSVNEEEVTIATINGEEKVKKTEKAEEDIESDYVGVPKEEDDSGKGISLDEEAGSIDYEHSMNAEAAGSIDYEHSVN 160

*P. cynomolgi* (PcyM_0732400) VGDSSENGEKVTSTASHGEAQA---RRADEGLTSDNADVAQEEDNSGEGISLVGEHLEDEDEHHGEAAAAGPINHEHHGE 157

*P. cynomolgi* (PCYB_073880) -------------------------------------------------------------------------------- 15

*P. coatneyi* (PCOAH_00015900) VDDSTANGEKVTIVTIDGEAQV---EKADEEVKGDNAGAPKEEDDSGTGISLNREAWVDEDEHYGDAYTG---------- 147

*P. fragile* (AK88_03613) EDDSSANDEKVTSASSSGEEEV---EKENEHVKSNIADALQQEDESNAGISLDGKNWEDEDEHIVEA--N---------- 145

*P. inui* (C922_04914) VDDSTANDEKFIVSIGHDGQVT--VEKAHEENTSDNAEVSHREDHSNAEVP----QWEDEDEN----------------- 137

*P. vivax* (PVX_087140) ANAPASIPDHHYGEANAPASIPDHHYGEA------------------------------------------NAPASIPDH 190

*P. vivax* (PVP01_0730000) ANAPASIPDHHYGEANAPASIPDYHYGEA------------------------------------------NAPASI--- 187

*P. malariae* (PmUG_07043100) --GNAAIGYNVFDNAPGNDEFTNAPDNDEFANAPGNDEFANSPGNAAP-------------------------------- 164

*P. knowlesi* (PKNH_0730000) AEAADSIDYEHSVNAEAADSID---YEHSM-------------------------NAEAAGSIDYEHSVNAEAADSIDYE 212

*P. knowlesi* (PKNOH_S06432100) AEAADSIDYEHSVNAEAADSID---YEHSM-------------------------NAEAAGSIDYEHSVNAEAADSIDYE 212

*P. cynomolgi* (PcyM_0732400) AAAAGPINHEHYGEAAAAGPINHAYYGEAAAAGPINHEYNGEAAAAGPINHAYYGEAAAAGPINHEYNGEAAAAGPINHA 237

*P. cynomolgi* (PCYB_073880) ----GPINHEHYGEAAAAGPINHAYYGEAAAAGPINHEYNGEAAAAG--------------------------------- 58

*P. coatneyi* (PCOAH_00015900) --PAAAMDHEHYGDASP---------------GPAAGMYDEQ-----------YGDA---------YTG---PAAAMDHE 187

*P. fragile* (AK88_03613) --SADLVNNEHVVEANQ--------------------------------------------------------ADLVNNE 167

*P. inui* (C922_04914) -------------------------YGTAGPAPAGGADYEED-------------------------------------- 154

*P. vivax* (PVX_087140) HYGEANAPASIPHYHYDEANAEG----------------------------PITHEHYGEANAPASIPDYHYGEANAAGP 242

*P. vivax* (PVP01_0730000) -----------PHYHYDEANAEG----------------------------PITHEHYGEANAPASIPDYHYGEANAAGP 228

*P. malariae* (PmUG_07043100) ---------------------------DYDEFANAPGNDEF-----------------------------------ANAP 182

*P. knowlesi* (PKNH_0730000) HSMNAEAAGSID---YEHSM-----------NAEAAGSIDYEHSMNAETAGSID-------------------------- 252

*P. knowlesi* (PKNOH_S06432100) HSMNAEAAGSID---YEHSM-----------NAEAAGSIDYEHSMNAETAGSID-------------------------- 252

*P. cynomolgi* (PcyM_0732400) YYDEAAAAGPINHAYYDEAAAAGPINHEYNGEAAAAGPINHEYYGPPGAAGPINHEYYGPAGAAGPINHEYNGEAAAAGP 317

*P. cynomolgi* (PCYB_073880) -------------------------------------------------------------------------------- 58

*P. coatneyi* (PCOAH_00015900) QYGDANPG-----------PAAGMYDEQYGDA----------YTGPAAA---MDHEQYGDANP-GPAAGMYD-------- 234

*P. fragile* (AK88_03613) HVVEDNAA-----------PAE---------------------------------------------------------- 178

*P. inui* (C922_04914) -----------------------------------------------NPTGPMHHEHYGAAA-----------QAPAGGP 176

*P. vivax* (PVX_087140) MPHEHEGGANPAGPTPHYHHAEANAPDSIPHYHYDADGA----------------------------------PPAGAPY 288

*P. vivax* (PVP01_0730000) MPH----------------------------YHYDADGA----------------------------------PPAGAPY 246

*P. malariae* (PmUG_07043100) GNDEFANSPGNAAPDYNEFANAPDNAAPDYNEFANAPGNAAPDYDKF--ANAPGNAAPDHGEHAGAS------------- 247

*P. knowlesi* (PKNH_0730000) ---------------------------------------------------------------YEHSMNAEAGASEGEEH 269

*P. knowlesi* (PKNOH_S06432100) ---------------------------------------------------------------YEHSMNAEAGASEGEEH 269

*P. cynomolgi* (PcyM_0732400) INHEYYGPAGAAGPINHEYNGEAAAAGPINHEYYGEAAAAGPMYDEHHGEA--GAAGPMYDEHYGEAGGAVPPPSEGGAH 395

*P. cynomolgi* (PCYB_073880) -----------------------------------------------------------------------------GAH 61

*P. coatneyi* (PCOAH_00015900) ---EHYGDASP-GPAAGMYDEQYGDASP---------GPAAGMYDEQYGDASPGPAAGMDHEHYGNAD-VVPPPAGGEAY 300

*P. fragile* (AK88_03613) -------------------DGAYTQTTQ---------------------------------------------------- 187

*P. inui* (C922_04914) ----DYGAVGPPPAQGVDYHEEYP-------------APAGGAY---HGAGNPG-------------------PAQGGEQ 217

*P. vivax* (PVX_087140) APNKLDEQAEDAFIRFFSQKPCITLPGEERSEKCNDAERGDNKEYDIKITYNEKEEHINRGENKCVNLNLNLNNGSPPSE 368

*P. vivax* (PVP01_0730000) APNKLDEQAEDAFIRFFSQKPCITLPGEERSEKCNDAERGDNKEYDIKITYNEKEEHINRGENKCVNLNLNLNNGSPPSE 326

*P. malariae* (PmUG_07043100) -------------------IIFNGSNESKDCNGEKNGN--IWRTECIFDDKERNNNINRKWNGKINFTINEKNAQ-E 302

*P. knowlesi* (PKNH_0730000) THKKFDGQSEDAFIRFFSPKSCITLPGEEASEKCKDVRGGNNSEYDIKVTYNEKEEHINRGENKCINLNIN--DGSPPSK 347

*P. knowlesi* (PKNOH_S06432100) THKKFDGQSEDAFIRFFSPKSCITLPGEEASEKCKDVRGGNNSEYDIKVTYNEKEEHINRGENKCINLNIN--DGSPPSK 347

*P. cynomolgi* (PcyM_0732400) TIKKLDEQTEGAFIRFFSPKSCITLPGEE---KCKDVQGENNQGYDIKVTYNEKEEYIKRGDNRCVNLNIN--DGSPPSK 470

*P. cynomolgi* (PCYB_073880) TIKKLDEQTEGAFIRFFSPKSCITLPGEE---KCKDVQGENNQGYDIKVTYNEKEEYIKRGDNRCVNLNIN--DGSPPSK 136

*P. coatneyi* (PCOAH_00015900) T-RKLDEQAEDAFIRFFSPKPCITLPGEEPSAKCKDMQKGNNKEYDIKVTYNEKEEHINRGDNKCVNLNIN--DGSPPSK 377

*P. fragile* (AK88_03613) ---------DEQAQDAFIRFFSPKPCIVLPGEEGSDKCKDVRGGNNKDYDIKVTYNEKEEHINRGENKCVNLNIN--DGSPPSK 260

*P. inui* (C922_04914) RGRKLDQQAHNAYIRFFSQKRCIHLPGEERSLNCRDMQGENDHQYDIKVTYNEKEEHINGHENKCLNLNIN--DGSPPSK 295

*P. vivax* (PVX_087140) DGPSNVF-INLSFVPNIPEEIINDFYAIIKRLKHMFEFMDPQEGTAPVEEVGSEQEVNMERAGSEEEANMERAGSEEEAN 447

*P. vivax* (PVP01_0730000) DGPSNVF-INLSFVPNIPEEIINDFYAIIKRLKHMFEFMDPQEGTAPVEEVGSEQEVNMERAGSEEEANMERAGSEEEAN 405

*P. malariae* (PmUG_07043100) VGHNMVFHLNISLIPGMQQEYLKNIYTVLTDLKKLLENAQKKEN. 347

*P. knowlesi* (PKNH_0730000) DGPSNIF-INLSLVPNIPEEIINDFYSIIKKLKTMFEIMEPEESNAQTEIIGSA. 401

*P. knowlesi* (PKNOH_S06432100) DGPSNIF-INLSLVPNIPEEIINDFYSIIKKLKTMFEIMEPEESNAQTEIIGSA. 401

*P. cynomolgi* (PcyM_0732400) DGPSNIF-INLSFVPNIPEEVINDFYSIIKKLKHMFEIVEPQENSVQPERIGGGTESA. 528

*P. cynomolgi* (PCYB_073880) DGPSNIF-INLSFVPNIPEEVINDFYSIIKKLKHMFEIVEPQENSVQPERIGGGTESA. 194

*P. coatneyi* (PCOAH_00015900) DGPSNIF-INLSFVPKIPEEVINDFYLIIKKLKHMFEIVEPEENNAQTEIRGT--------V. 431

*P. fragile* (AK88_03613) DGPSNIF-INLSFVPNIPEEIINDFYHIIKRLKFMFEVMEPEENSVPSEMAGSAQEAKEEML. 322

*P. inui* (C922_04914) DGPSNIF-INLSFVPNIPEEIINDFYAIIGKLKYMFEIVEPEQNSVPAEQNSVPTEQNSVPAEQNSVPTEQNSVPTEQNS 374

*P. vivax* (PVX_087140) MERAGSEEEAKR---K. 461

*P. vivax* (PVP01_0730000) MERAGSEEEAKR---K. 419

*P. malariae* (PmUG_07043100) 347

*P. knowlesi* (PKNH_0730000) 401

*P. knowlesi* (PKNOH_S06432100) 401

*P. cynomolgi* (PcyM_0732400) 528

*P. cynomolgi* (PCYB_073880) 194

*P. coatneyi* (PCOAH_00015900) 431

*P. fragile* (AK88_03613) 322

*P. inui* (C922_04914) VPTEQNSVPADRVGIE. 391
